# Effect of Methoxy Substituents on the Activation Barriers of the Glutathione Peroxidase-Like Mechanism of an Aromatic Cyclic Seleninate

**DOI:** 10.3390/molecules200610244

**Published:** 2015-06-03

**Authors:** Craig A. Bayse, Ashley L. Shoaf

**Affiliations:** Department of Chemistry and Biochemistry, Old Dominion University, Hampton Boulevard, Norfolk, VA 23529, USA; E-Mail: ashoa001@odu.edu

**Keywords:** organoselenium compounds, antioxidants, glutathione peroxidase, density functional theory

## Abstract

Density functional theory (DFT) models including explicit water molecules have been used to model the redox scavenging mechanism of aromatic cyclic seleninates. Experimental studies have shown that methoxy substitutions affect the rate of scavenging of reactive oxygen species differently depending upon the position. Activities are enhanced in the *para* position, unaffected in the *meta*, and decreased in the *ortho*. DFT calculations show that the activation barrier for the oxidation of the selenenyl sulfide, a proposed key intermediate, is higher for the *ortho* methoxy derivative than for other positions, consistent with the low experimental conversion rate.

## 1. Introduction

Organoselenium compounds have been extensively studied for their ability to mimic the activity of the antioxidant selenoprotein glutathione peroxidase (GPx) [1,2,3,4,5,6]. These synthetic analogues may be important for the prevention and treatment of various illnesses linked to excess reactive oxygen species such as cancer and cardiovascular disease. In early studies, interactions between selenium and a nitrogen-containing group was believed to be important for a highly active redox catalyst until Wirth showed in an article in *Molecules* that a series of diselenides with intramolecular Se···O interactions could also mimic GPx [7]. Over the years, Back and others have reported additional GPx mimics containing either Se···O interactions or Se–O bonds such as the cyclic seleninates **1** [8,9,10,11,12,13,14].

Many organoselenium GPx mimics have complex mechanisms which are challenging to monitor experimentally. We have taken a computational approach using density functional theory (DFT) to assist in the deciphering of these mechanisms to examine bonding and mechanistic aspects of these processes [15,16]. Specifically, we have performed DFT studies of the redox mechanisms of sulfur and selenium compounds using the solvent-assisted proton exchange (SAPE) method [15,17,18,19,20,21]. These models incorporate explicit solvent molecules (specifically water) in order to provide a first approximation to the role of bulk solvent in proton transfer processes. DFT-SAPE models have been very successful in reproducing the experimental barriers and trends in activities of these biochalcogen compounds. Many of the processes involved in the mechanisms of GPx mimics are nucleophilic attacks coupled to protonation/deprotonation of the nucleophile and leaving group. Direct transfer of this proton would require a four-centered transition state that is highly strained and unlikely to occur in solution. SAPE models position the reactants in the correct orientation for backside attack and add a hydrogen-bonded network of water molecules to facilitate the shifting of the proton between the reactants and products. Because the mechanism is modeled based upon the expected pathway in solution, SAPE transition states are significantly lower in energy than those forcing direct transfer and allow for more mechanistic insight. As a result, SAPE models are becoming an important tool for modeling of solution-phase processes using conventional DFT.

**Scheme 1:**
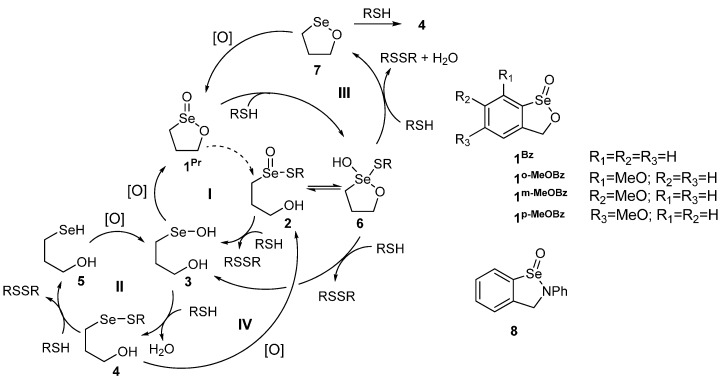
Mechanisms for ROS scavenging for five-membered cyclic seleninates such as **1^Pr^** and **1^Bz^**.

We recently reported an extensive DFT-SAPE study of the pathways available for redox scavenging by **1^Pr^** [21]. Four potential pathways (Scheme 1) were explored for this reaction: a modified version of the originally proposed mechanism (**I**: **1**→**6**→**2**→**3**→**1**), the mechanism analogous to GPx (**II**: **3**→**4**→**5**→**3**), a proposed cyclic selenenate cycle (**III**: **1**→**6**→**7**→**1**) and an alternate mechanism based upon the oxidation of **4** (**IV**: **4**→**2**→**3**→**4**). Back’s original mechanism **I** was modified to account for the large barrier calculated for the direct conversion of **1** to the seleninyl sulfide **2**. Instead, a lower energy pathway through a selenurane intermediate **6** which rearranges to **2** was found. One of the key problems with mechanism **I** is the re-formation of the cyclic seleninate **1** through oxidation of the selenenic acid **3**. Our calculations suggest that this is a relatively high barrier process that would face significant competition from conversion to the selenenyl sulfide **4**, an intermediate believed to be a dead end in many GPx-like mechanisms because the activation barrier for reduction to the selenol **5** is prohibitively high. The GPx-like pathway **II** is well-known to have a high barrier for regeneration of the selenol (**4**→**5**). The cyclic selenenate cycle **III** was considered as an alternative to **I** or **II**, but the rate-determining step was found to be the reduction of **6** to **7**, inconsistent with Hammett plot studies showing that oxidation of Se should be rate determining [12].

We proposed pathway **IV** after DFT-SAPE studies showed that mechanisms **I**-**III** each involves either a high barrier for further reaction of **4** or a pathway to **4** with a lower activation barrier than the rate-determining step for the mechanism [21]. Similarly, we suggested that the slow ROS scavenging by ebselen **8** and related derivatives with simple thiols could also be due to oxidation of the selenenyl sulfide as the rate-determining step [18]. With dithiols, glutathione or thiols with internal S···N interactions, ebselen was proposed to operate through a GPx-like cycle due to either the effects of conversion to a unimolecular process (dithiols) or intramolecular interactions upon the reduction of the selenenyl sulfide [18,22].

Back has shown that the aromatic cyclic seleninate **1^Bz^** has lower GPx-like activity than **1^Pr^** [12]. An analysis of the effect of electron-donating and -withdrawing groups through a Hammett plot analysis suggested that oxidation of selenium occurs at the rate-determining step [10]. A later study of the effect of methoxy substitution showed that *para* substitution enhances activity due to the mesomeric effect while *meta* substituents did not affect activity [12]. *Ortho* substitutions show a significant reduction in activity, possibly due to steric or coordination effects. Surprisingly, this result contrasts with kinetics studies by Mugesh where *ortho*-methoxy groups enhance the activity of amine-based GPx mimics [23]. In these compounds, strong Se···N interactions increase the sterics and/or electrophilicity of the selenium of the selenenyl sulfide intermediate to prefer catalytically unproductive thiol exchange over reduction to the selenol [2,22,24]. The *ortho*-methoxy group interferes with this interaction to lower the barrier for selenol regeneration [20]. It is not clear why this substitution is problematic for the cyclic seleninates, but the Se···O interaction is generally much weaker than a typical Se···N interaction such that steric interference by the methoxy group may be more significant in derivatives of **1** [20]. The *o*,*p*-dimethoxy derivative of **1^Bz^** was monitored by HPLC to show the accumulation of the selenenyl sulfide **4**. These results may be consistent with our conclusions about the importance of oxidation of **4** to the ROS scavenging of **1** and other organoselenium GPx mimics and suggests that *ortho* substitution may increase the barrier to oxidation of **4** to prevent catalytic activity. Therefore, in this paper, we revisit our SAPE study of cyclic seleninates to explore the effect of methoxy substituents on the key activation barriers of the GPx-like mechanism of **1^Bz^**. 

## 2. Results and Discussion

**Figure 1:**
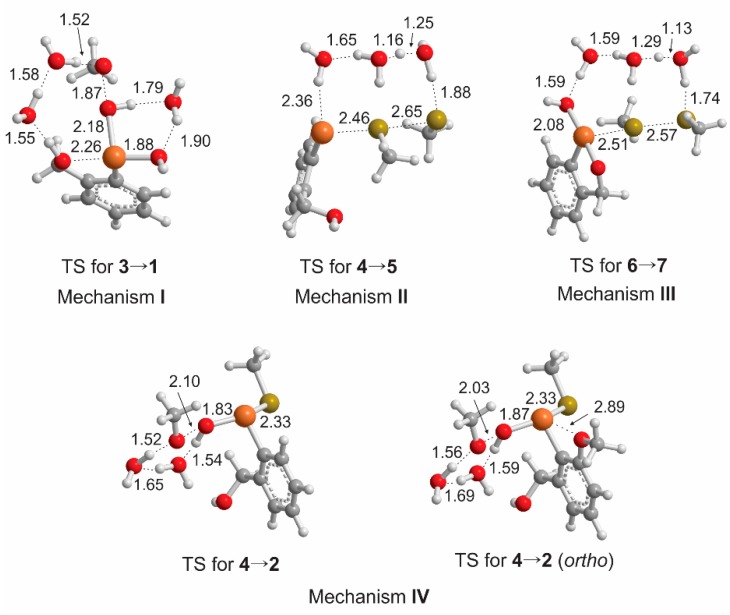
Selected bond distances for the transition states of the rate determining steps in proposed mechanism **I**–**IV** for **1^Bz^**.

SAPE models for the proposed mechanism for ROS scavenging by aromatic cyclic seleninates were based upon our previous study on **1^Pr^** [21], replacing the 3-hydroxypropyl group with a benzyl alcohol backbone. Methyl thiol (MeSH) and methyl hydrogen peroxide (MeOOH) were used as the model thiol and oxidant, respectively. Activation barriers of the *o*-, *m*-, and *p*-methoxy derivatives were also determined for comparison to Back’s experimental half-lives [12] for the steps involving oxidation of the selenium center only. Structures of the reactant, transition state and product SAPE complexes for **1^Bz^** were optimized using the same water network [21] with the same hydrogen bond connectivity as in the study of **1^Pr^** for consistency within the various models. As a result, the structural parameters for the SAPE models for **1^Bz^** are similar to those for **1^Pr^** and only selected structures are presented in this paper (Figure 1). The direct reaction of **1^Bz^** with MeSH was omitted because this reaction appears to be through the initial formation of selenurane **6** which isomerizes to **2** through a barrier that is expected to be small [21,25].

Most of the activation barriers (Table 1) vary only slightly from **1^Pr^**, with the exception of the oxidation of **5** and the reduction of **6** to the cyclic selenenate **7**. For the original cycle **I**, the oxidation and re-cyclization of **3** to re-form **1** is slightly lower than in **1^Pr^**, and the conversion to the selenurane **6** is slightly less favorable. In the GPx-like cycle **II**, the barrier to oxidation of the selenol **5** is lower than for the alkyl derivative and more similar to the unsubstituted benzeneselenol (19.1 kcal/mol) [17]. However, the rate-determining step (**4**→**5**) is comparable to that in **1^Pr^** and has a slightly higher activation barrier using the PCM solvation correction for CH_2_Cl_2_ (the experimental solvent was 95:5 CH_2_Cl_2_:CH_3_OH) rather than water (22.5 kcal/mol [20]). The reduction of **6** to **7** in mechanism **III** is 4.9 kcal/mol more favorable than for the 3-hydroxypropyl derivative, but is still not low enough to compete with rapid rearrangement of **6** to **2** and further reaction through mechanisms **I**, **II**, or **IV**. Therefore, mechanism **III** appears unlikely to contribute to redox scavenging. Even if **7** were accessible, it would be preferably reduced to **4** over oxidation to **1** given the low barrier for **7**→**4**. 

**Table 1:** DFT-SAPE(mPW1PW91) activation barriers and energies of reaction for proposed steps in the redox catalysis of **1^Bz^**. Values for **1^Pr^** are listed in parenthesis for comparison.

Step	∆G^‡^ + ∆G_solv_, kcal/mol	∆G + ∆G_solv_, kcal/mol
**2→3**	16.2 (16.9)	−14.4 (−7.8)
**3→1**	18.9 (19.7)	−44.0 (−47.5)
**3→4**	8.1 (8.4)	−29.9 (−34.5)
**4→5**	25.1 (25.2)	4.6 (9.7)
**5→3**	20.0 (24.5)	−59.4 (−59.4)
**1→6**	9.2 (8.4)	−5.8 (−1.0)
**6→3**	16.3 (14.1)	−15.8 (−24.9)
**6→7**	12.0 (16.9)	−21.6 (−22.4)
**7→1**	11.1 (12.2)	−48.6 (−55.6)
**7→4**	4.4 (3.6)	−20.8 (−23.7)
**4→2**	23.1 (22.3)	−33.2 (−36.1)

Each of the barriers obtained for mechanisms **I**–**III** for **1^Bz^** are consistent with our conclusions for **1^Pr^**. Each contains either a bottleneck at **4** or a low-energy competing pathway to **4**, suggesting that a mechanism involving the oxidation of **4** to **2** is needed to allow for cycling of the catalyst. In **1^Bz^**, the activation barriers for **4**→**2** are slightly higher than that for **1^Pr^**, consistent with the lower activity of the aromatic derivative. As determined for **1^Pr^**, mechanism **IV** is the lowest energy pathway for GPx-like activity calculated using DFT-SAPE. 

Because Hammett plots suggest that oxidation of the selenium center is important to the rate-determining step [10], DFT-SAPE models were generated for the oxidation steps (**3**→**1**, **5**→**3**, **7**→**1**, **4**→**2**) substituted at the *para*, *meta* and *ortho* positions (Table 2). For the steps involving oxidation to or from **3**, the activation barriers do not vary significantly with substitution and are slightly higher in energy than **1^Bz^**. The activation barriers for the remaining oxidations **7**→**1** and **4**→**2** follow the general trend of the experimental half-lives (**1^p-MeOBz^** < **1^Bz^** ≈ **1^m-MeOBz^** < **1^o-MeOBz^**), although the former step is unlikely to be a factor in the overall mechanism because mechanism **III** is not clearly accessible and **7**→**1** is not its rate-determining step. In our viewpoint, the DFT-SAPE and experimental results point to the selenenyl sulfide **4** as the key intermediate in these GPx mimics because all proposed mechanisms have low barrier pathways to this intermediate. The barriers for **4**→**2** (Table 1 and Table 2) are consistent with both the low activity of the species and the proposal of this step as key to the mechanism of this type of GPx mimics, especially for the *ortho* methoxy derivative of **4** where a close interaction between Se and the methoxy group destabilizes the transition state for oxidation (Figure 1). The significantly higher barrier for the *ortho* derivative is in agreement with the deactivation of the *o*,*p*-dimethoxy derivative catalyst to **4**. Cycling of the catalyst from **4** is either through the reduction to the selenol (**4**→**5**) or the oxidation to the seleninyl sulfide (**4**→**2**). The reduction step **4**→**5** is a known bottleneck in GPx-like cycles and retains a high barrier in **1^Bz^** (25.1 kcal/mol). In the *ortho* substituted derivatives, redox cycling is slow due to the accumulation of **4** due to the generally high barrier to reduction to **5** and the closing of the oxidation pathway to **2**. 

**Table 2:** DFT-SAPE(mPW1PW91) activation barriers and energies of reaction for oxidation steps in the redox catalysis of methoxy-substituted **1^Ph^**.

		∆G^‡^ + ∆G_solv_, kcal/mol	∆G + ∆G_solv_, kcal/mol
**3→1**	*para*	20.1	−45.8
	*meta*	20.6	−44.6
	*ortho*	20.5	−47.2
**5→3**	*para*	21.0	−58.4
	*meta*	21.0	−58.9
	*ortho*	21.0	−54.1
**7→1**	*para*	8.2	−52.4
	*meta*	9.4	−51.4
	*ortho*	14.6	−52.5
**4→2**	*para*	21.8	−34.9
	*meta*	23.2	−35.2
	*ortho*	25.0	−32.5

## 3. Experimental Section

Calculations were performed using Gaussian09 and the mPW1PW91 exchange correlation functional using the same procedures as described in previous DFT-SAPE calculations of biochalcogen systems [15,16,17,18,19,20,21]. Readers are referred to these previous studies for a more detailed discussion of the design of SAPE-type models as used in these studies. Experimental studies of **1^Bz^** were performed in 95:5 CH_2_Cl_2_/MeOH with the addition of aqueous solutions of hydrogen peroxide and it is assumed that there are sufficient protic solvent molecules to facilitate proton exchange by clustering about the polar functional groups of the reactants. For simplicity, water is used to represent all of the protic solvent molecules. The design of the specific SAPE models for the steps in Scheme 1 for **1^Bz^** and its monosubstituted methoxy derivatives was based upon the previously published calculations [21] of the mechanism of **1^Pr^** and used 2–4 water molecules to facilitate the transfer of a proton between the reactant and product. The mPW1PW91 exchange correlation functional was shown to give similar barriers and structures for a representative SAPE model relative to MP2 [15]. Hydrocarbon fragments were represented by the Dunning double-ζ basis set with polarization functions added to carbon. Oxygen and hydrogens bonded to non-carbon heavy atoms were represented by the Dunning triple-ζ basis set. Diffuse functions were added to oxygen, sulfur and selenium. Transition states were characterized by an imaginary mode whose atomic displacements were consistent with motion along the reaction coordinate for the specific step being modeled. Solvation corrections in dichloromethane using the IEF-PCM model were included in the free energy calculations.

## 4. Conclusions

The inclusion of explicit water molecules through DFT-SAPE models is an effective means of representing the role of bulk solvent in reactions requiring an exchange of a proton between reactant and product. DFT-SAPE modeling of the GPx-like mechanism of **1^Bz^** are in agreement with its slower activity relative to **1^Pr^** assuming that redox scavenging occurs primarily through mechanism **IV** with **4**→**2** as the rate-determining step. Previous DFT-SAPE studies point to **4** as an important intermediate because all proposed mechanisms either include **4** in the rate-determining step or a low-energy competing pathway to **4**. Activation barriers calculated for the methoxy derivatives of **4**→**2** agree with the observed trend for the half-lives of these species (**1^p-MeOBz^** < **1^Bz^** ≈ **1^m-MeOBz^** < **1^o-MeOBz^**) [12], further suggesting that oxidation of **4** is a key step in the mechanisms of this reaction. Other oxidation steps either do not differentiate the activation barriers for different methoxy positions or are not rate-determining steps in their mechanisms. The accumulation of **4** in the experimental redox scavenging by the *o*,*p*-dimethoxy derivative is consistent with *ortho* substitution increasing its activation barrier for oxidation.
